# Antibody-drug conjugates: beyond current approvals and potential future strategies

**DOI:** 10.37349/etat.2022.00082

**Published:** 2022-04-28

**Authors:** Siddharth Menon, Sagun Parakh, Andrew M. Scott, Hui K. Gan

**Affiliations:** 1 Olivia Newton-John Cancer Centre at Austin Health, Olivia Newton-John Cancer Wellness & Research Centre, Heidelberg Victoria 3084, Australia; 2 College of Science, Health and Engineering, La Trobe University, Melbourne Victoria 3086, Australia; Oncology Institute of Southern Switzerland, Switzerland

**Keywords:** Antibody-drug conjugates, immunotherapy, tumour microenvironment

## Abstract

The recent approvals for antibody-drug conjugates (ADCs) in multiple malignancies in recent years have fuelled the ongoing development of this class of drugs. These novel agents combine the benefits of high specific targeting of oncogenic cell surface antigens with the additional cell kill from high potency cytotoxic payloads, thus achieving wider therapeutic windows. This review will summarise the clinical activity of ADCs in tumour types not covered elsewhere in this issue, such as gastrointestinal (GI) and genitourinary (GU) cancers and glioblastoma (GBM). In addition to the ongoing clinical testing of existing ADCs, there is substantial preclinical and early phase testing of newer ADCs or ADC incorporating strategies. This review will provide selected insights into such future development, focusing on the development of novel ADCs against new antigen targets in the tumour microenvironment (TME) and combination of ADCs with immuno-oncology (IO) agents.

## Introduction

The anti-cancer armamentarium is rapidly expanding with the development of novel targeted therapies, immune checkpoint inhibitors (ICIs), and antibody-drug conjugates (ADCs) [1–3]. ADCs consist of a monoclonal antibody specific to a cell surface target antigen which is conjugated to a cytotoxin or “payload” via a chemical linker, thereby providing selective delivery of a highly potent cytotoxic agent and effectively widening the therapeutic index of the drug [4–6]. The preclinical and clinical landscape of ADC development is in an exciting phase with a flurry of recent approvals in advanced solid tumours. The essential preclinical criteria for the development of a successful ADC are a selection of a valid target antigen with limited or no expression on healthy tissue, antibody, and linker optimisation and lastly efficient binding, internalization, and ultimately release of the payload [7]. Combining the specificity and activity of antibodies against targets on the cancer cell with a potent cytotoxic payload is a highly attractive therapeutic strategy, with the exploration of conjugating antibodies with toxins dating back to the 1970s [8]. Since then, significant strides have been made with the discovery of newer cancer-specific target antigens, development of novel cytotoxic payloads with potency in the picomolar range, and improvements in linker design; all of which have contributed to the growing success of ADCs [6, 9–13]. There are currently 11 Food and Drug Administration (FDA) approved ADCs for solid organ and hematological malignancies and over 80 ADCs in development [6, 7, 14–16]. Further benefits are anticipated if we can address issues of poor tumour penetration, appropriate biomarker use, tumour heterogeneity, toxicity and overcoming resistance mechanisms [17].

In this paper, we will first review the recent progress made by ADCs, focusing on gastrointestinal (GI) and genitourinary (GU) cancers as well as glioblastoma (GBM). We will then use a tumour agnostic approach to discuss future directions of ADC development, highlighting the most promising new ADCs in development and novel strategies for ADC integration with other treatments. Specifically, we will provide a review of the scientific rationale and most recent evidence for combination strategies with immuno-oncology (IO) agents, and the scope for targeting the tumour microenvironment (TME) using ADCs.

## ADCs in GI malignancies

Cancers of the upper and lower GI tract are a group of anatomically and molecularly heterogeneous diseases, possessing a wide variety of genetic aberrations, some of which are important therapeutic targets [18–20]. The addition of antibodies targeting human epidermal growth factor 2 receptor (HER2), vascular endothelial growth factor (VEGF), and epidermal growth factor receptor (EGFR) to conventional cytotoxic chemotherapy have resulted in superior outcomes for patients with oesophageal, gastric, and colorectal cancers (CRCs) [21–25]. Nevertheless, there is plenty of scope for improving therapeutic results in the field, with the potential of ADC integration in the treatment paradigm yet to be realised. Of all relevant targets, HER2-targeting ADCs have made the most progress in the field to date.

### Gastric and gastro-oesophageal cancers

Ado-trastuzumab emtansine (T-DM1; Kadcycla^®^, Genentech/Roche) is a humanized anti-HER2 antibody conjugated to a cytotoxic drug maytansinoid 1 (DM1) payload via a non-cleavable linker [26]. In 2013, T-DM1 became the first approved ADC in solid tumours for use in patients with HER2 overexpressing metastatic breast cancer who have previously received the anti-HER2 antibody trastuzumab (Herceptin^®^, Genentech) and chemotherapy [27]. Following the success of T-DM1 in patients with HER2-positive breast cancer progressing on dual blockade with HER2 targeting antibodies trastuzumab and pertuzumab [28], the trastuzumab emtansine *vs.* taxane use for previously treated HER2-positive locally advanced or metastatic (la/m) gastric or gastro-oesophageal junction (GOJ) adenocarcinoma (GATSBY) trial evaluated the drug in a gastric cancer setting. The phase II/III randomised, an open-label adaptive study assessed two dosing regimens of T-DM1 *vs.* taxane chemotherapy in patients with advanced HER2-positive gastric cancer progressing on or after 1st line therapy [29], with overall survival (OS) as the endpoint. The trial failed to meet its primary endpoint, with the OS inferior in the optimal dose T-DM1 arm compared to chemotherapy [7.9 months *vs.* 8.6 months, hazard ratio (HR) 1.15, one-sided *P* = 0.86]. No new safety signals were observed with the ADC and the rates of ≥ grade 3 treatment related adverse events (AEs; TRAEs) were similar in the ADC and chemotherapy arms.

Trastuzumab deruxtecan (DXd; T-DXd; DS-8201a: Enhertu^®^) is composed of a humanized anti-HER2 antibody conjugated to a topoisomerase I inhibitor via a cleavable peptide linker [30]. T-DXd has a higher drug to antibody ratio (DAR) than T-DM1 (8 *vs.* 3.5) and in preclinical gastric cancer models with HER2 overexpression demonstrated greater anti-tumour activity compared to T-DM1 [31–33]. The DESTINY-Gastric01 phase II trial in patients with HER2 positive gastric and GOJ cancers who had received at least two lines of prior treatment (including trastuzumab) demonstrated significantly superior objective response rates (ORRs) and OS with T-DXd therapy compared to physician’s choice chemotherapy (51% *vs.* 14%, *P* < 0.001 and 12.5 months *vs.* 8.4 months; HR for death, 0.59, *P* = 0.01 respectively) [34]. In a post-hoc exploratory biomarker analysis of the DESTINY-Gastric01 study, unlike in the corresponding breast cancer trial [35], clinical benefit was not found to be significant in *HER2* low disease. In fact, high level of *HER2* as defined by tissue messenger RNA (mRNA) expression or plasma *HER2* copy number was predictive of response in this analysis [36]. Notable toxicities associated with the ADC included hematological events and interstitial lung disease (ILD). Grade 3 or higher rates of neutropenia (51% *vs*. 24%), anemia (38% *vs.* 23%) and decreased white-cell count (21% *vs.* 11%) were greater in the T-DXd than in the chemotherapy arm and required appropriate dose modifications. The clinically concerning AE of ILD that has been reported in other T-DXd trials [35, 37] was reported in 10% of patients, with two grade 3 and one grade 4 event. T-DXd received FDA approval in January 2021 for patients with metastatic HER2-positive gastric or GOJ adenocarcinoma who have received a prior trastuzumab-based regimen [38]. Of note, the dose used in the gastric cancer trial of 6.4 mg per kilogram is higher than the approved dose of 5.4 mg per kilogram in breast cancer—a trial which reported an ILD rate of 13.6% and four (2.2%) related deaths [34, 35]. Currently, T-DXd is being evaluated in a phase II trial in patients with HER2-positive gastric cancer in an earlier line of therapy i.e., progressed on or after trastuzumab treatment (phase II DESTINY-Gastric02, NCT04014075) as well as in combination with chemotherapy, immunotherapy and trastuzumab (phase Ib/II DESTINY-Gastric03, NCT04379596).

Another HER2 targeting ADC, disitamab vedotin (RC48) which consists of an anti-HER2 antibody (hertuzumab) conjugated to a monomethyl auristatin E (MMAE) payload via a cleavable linker has been shown to be well tolerated and demonstrated promising clinical activity in phase I studies [39, 40]. Preclinical studies including enzyme-linked immunosorbent assay (ELISA)-based binding assays of hertuzumab have previously demonstrated higher affinity to HER2 compared to trastuzumab [41]. A single-arm phase II trial (NCT03556345) in patients reported clinically meaningful benefit with response rates of approximately 18% and median OS (mOS) of 7.6 months in a heavily pre-treated HER2 positive [immunohistochemistry (IHC)2^+^ or IHC3^+^] gastric/GOJ cancer population [42]. The phase III trial (NCT04714190) comparing RC48-ADC to physician’s choice chemotherapy in the 3rd and later line treatment of *HER2* overexpressing advanced gastric cancer is currently recruiting.

### CRCs

Approximately 5% of patients with CRC harbour HER2 alterations, predominantly amplification and overexpression, thus representing an important oncogenic target [43, 44]. Targeting HER2 using monoclonal antibodies and tyrosine kinase inhibitors (TKIs) has been investigated in previous trials such as the HER2 amplification for colo-rectal cancer enhanced stratification trial (HERACLES-A; NCT03225937) and the MyPathway solid tumour basket study. The former was a proof-of-concept phase II trial of the combination of trastuzumab and the dual EGFR/HER2 targeting TKI lapatinib while the latter was a phase IIa study assessing trastuzumab in combination with pertuzumab [45, 46]. These studies suggested that the combinations were active, yielding an ORR of 30%, and 32% respectively with acceptable safety in treatment-refractory HER2-positive advanced CRC. Unfortunately, the HERACLES-B study, a single-arm phase II trial assessing the combination of pertuzumab and T-DM1 in rat sarcoma (RAS)/v-raf murine sarcoma viral oncogene homolog B1 (BRAF) wild type, *HER2* overexpressing metastatic CRC (mCRC) failed to meet its primary endpoint with an ORR of 10% [47]. In order to assess the potential activity of T-DM1 in patients exposed to prior HER2 targeted therapy, the phase II HERACLES-RESCUE (NCT03418558) trial is evaluating T-DM1 in *HER2* amplified mCRC patients progressing within the HERACLES-A trial [48].

In keeping with recent encouraging efficacy data in breast, gastric and non-small cell lung cancer (SCLC; NSCLC) [34, 35, 37], T-DXd was also evaluated in an open-label phase II trial, DESTINY-CRC01, enrolling patients with HER2 overexpressing mCRC or unresectable CRC, following progression after at least 2 lines of prior—including 30% who had received prior HER2 targeting treatment [49]. Patients with HER2 expression were grouped into 3 cohorts—IHC3^+^/IHC2^+^ and *in situ* hybridization (ISH^+^; cohort A: HER high), IHC2^+^ and ISH^–^, and lastly IHC1^+^ (cohorts B and C, respectively: HER2 low). In this trial, at a median follow up of 27 weeks, 45% of the 53 patients in cohort A experienced a confirmed objective tumour response, including 1 patient with a complete response (CR). There were no responses in cohorts B and C although there were far fewer patients as patient accrual to these cohorts was ongoing at the time of analysis. The main safety concern associated with T-DXd of ILD was seen in 6% of all patients including two grade 5 events, which was lower than the rates seen in breast, lung, and gastric cancer trials [50]. In a heavily pre-treated population, T-Dxd demonstrated strong and durable responses in *HER2* overexpressing tumours as monotherapy across subgroups and further testing in larger trials of single agent and combination therapy are warranted.

Carcinoembryonic antigen (CEA)-related cell adhesion molecule 5 (CEACAM5), belonging the CEA family is a cell adhesion molecule highly expressed in carcinomas including those of the GI tract [51]. Labetuzumab govitecan (LG; IMMU-130) is an ADC consisting of an antibody targeting CEACAM5 conjugated to SN-38, the topoisomerase-I inhibitor and active metabolite of irinotecan [52]. In a phase I/II trial assessing safety and tolerability in 2 dosing schedules and efficacy, LG was assessed in a heavily pre-treated mCRC population demonstrating some activity—while only one patient of the 72 response evaluable participants had a partial response by the response evaluation criteria in solid tumours (RECIST) criteria, 38 patients had a decrease in tumour burden as well as plasma CEA levels. The clinical benefit rate (partial response combined with stable disease) > 4 months was 29% [53]. The drug was also found to have an acceptable safety profile— the most frequent grade ≥ 3 events included neutropenia (16%), anemia (9%) and diarrhoea (7%). While plans for future development of LG is unclear, the ADC could feature in future monotherapy or combination studies in mCRC [54].

## ADCs in GU malignancies

The management of la/m urothelial cancer (UC; mUC; la/mUC) is rapidly evolving, with the recent approval of multiple ICIs as well as fibroblast growth factor receptor (FGFR) targeted therapy [55]. However, treatment resistance remains an issue and as such, there has been growing interest in the use of ADCs in these malignancies that has in fact translated into some successful approvals. Nectin-4, trophoblast cell surface antigen 2 (Trop-2), and HER2 have all been assessed as promising targets in UC, with ADCs targeting Nectin-4 and Trop-2 gaining FDA approval in the last year. Treatment of clear cell (cc) metastatic renal cell carcinoma (RCC; ccRCC; cc-mRCC) has also undergone paradigm changing treatment strategies in the last few years. The combination of two ICIs, or one of ICI and one of several newer TKIs targeting VEGF/VEGF receptor (VEGFR) has in multiple trials significantly outperformed single agent TKI therapy, which has been the cornerstone of management for several years [56]. The ectonucleotide pyrophosphatase/phosphodiesterase 3 (ENPP3) antigen and T cell immunoglobulin mucin-1 (TIM-1) have been studied in early phase clinical trials as druggable ADC targets most recently. The management of prostate cancer has previously mostly relied on androgen deprivation, chemotherapy and novel hormonal agents have also borne witness to improvements in survival in the past decade. However, castration resistance and ultimately failure of chemotherapy and anti-androgen agents necessitate novel targets and treatments [57], including potentially other targeted therapeutics. Prostate-specific membrane antigen (PSMA), six-transmembrane epithelial antigen of the prostate 1 (STEAP1) and Trop-2 have all been subjects of early clinical ADC development.

### UC

Nectin-4 is a transmembrane adhesion molecule found to be overexpressed in several cancers, including in 60–80% of UC patients, where it is implicated in tumorigenesis, metastasis, and disease recurrence [58, 59]. Enfortumab vedotin (EV) consists of a monoclonal antibody targeting Nectin-4, linked to an MMAE payload and was clinically first assessed in the EV-101 trial. This phase I dose escalation/expansion study of patients with la/mUC treated with ≥ 1 lines of chemotherapy or ineligible for cisplatin demonstrated an encouraging ORR of 43% [60, 61]. The phase II EV-201 single arm study followed, assessing efficacy of EV in patients who had prior ICI treatment and also included a cohort of cisplatin ineligible patients [62]. Reported in May 2021, this trial reported a confirmed ORR of 52% amongst the 89 patients who received treatment at the 2018 data cut off including a 20% CR rate and median duration of response (mDOR) of 10.9 months [95% confidence interval (CI) 5.78 months–not reached]. Grade 3 or higher TRAEs occurred in 55% of the patient population and included neutropenia (9%), rash (8%) and fatigue (7%). There were 4 deaths (4%) that were attributed to study drug—one each from acute kidney failure, metabolic acidosis, multiorgan dysfunction, and pneumonitis. Peripheral neuropathy occurred in 54% of the subjects but was mostly lower grade. Full FDA approval was granted to EV on July 9, 2021 [63] following the results of the confirmatory EV-301 phase III trial demonstrating an OS benefit of approximately 4 months (12.9 months *vs.* 9 months, HR = 0.7; *P* = 0.001) in patients with la/mUC who had received platinum-based chemotherapy and prior ICI treatment *vs.* physician’s choice of chemotherapy [64]. The rates of grade ≥ 3 TRAEs were approximately 50% in both groups with the EV group reflecting similar AEs seen in the phase II trial—rash (7%), fatigue (6.4%), neutropenia (6.1%) and neuropathy (3.7%). TRAEs resulting death occurred in 7 (0.1%) patients in the EV arm and in 3 (0.0%) in the chemotherapy group.

Another cell adhesion molecule widely expressed in multiple solid organ cancers and implicated in tumour invasiveness and poor survival is the Trop-2 [65]. Sacituzumab govitecan (SG) consists of an anti-Trop-2 monoclonal antibody linked to the topoisomerase inhibitor SN-38 and has already received accelerated FDA approval for use in triple negative advanced breast cancer [66]. Accelerated approval for SG in mUC in 2021 [67] came from the multi-cohort phase II open-label study of SG in patients with metastatic urothelial carcinoma progressing after platinum-based chemotherapy and checkpoint inhibitors (TROPHY-U-01) investigating the ADC in a similar setting to the EV-301 trial. The bladder cancer cohort had 113 patients, with SG yielding an overall response rate of 27% with an mDOR of 7.2 months [68] which was lower than reported for in the phase II EV trial, EV-201 [62]. The grade 3 or higher TRAE rate in the phase II and 3 EV trials was approximately 51%, with myelosuppression and skin reactions being the most clinically impactful [62, 64]. In the TROPHY trial, grade 3 and higher TRAEs attributed to SG occurred in 55% of patient with neutropenia and GI toxicity being the most common [68].

The reported rates of *HER2* overexpression in bladder cancer vary significantly in the literature ranging from 0–80% and have been shown to be associated with higher grade of disease and a worse prognosis [69]. The HER2 targeting ADC RC48 was evaluated in a single arm phase II efficacy study (NCT03507166) in patients with la/mUC with HER2 overexpression (IHC2^+^/IHC3^+^) who had failed at least 1 line of systemic treatment [70]. Of 133 patients screened, 43 were enrolled and were included in the analysis at early termination of recruitment due to a prespecified ORR that was met (66.7% in the first 30 evaluable patients). Across all evaluable patients, the confirmed ORR was 51%—although 88% of all participants experienced some degree of tumour reduction—the mDOR was 6.9 months (95% CI, 4.7–10.8 months) and median progression free survival (PFS; mPFS) 6.9 months (95% CI, 5.6–8.9 months). Within specific subgroups, the ORR was 50% in patients with ≥ 2 lines of treatment, 52% in patients who had one prior line of treatment and 75% (8 patients) in those who had received prior immune checkpoint blockade (ICB) therapy. Tolerability was favourable with the most common grade 3 TRAEs being hypoesthesia (23%) and neutropenia (14%), while there were no grade 4 or 5 TRAEs. It is unclear what the future plans for RC48 are.

### Prostate cancer

PSMA is a membrane glycoprotein selectively overexpressed in prostate cancer, targeting which has been the subject of several new preclinical and clinical investigational agents [71, 72]. A PSMA targeting ADC consisting of a human immunoglobulin G1 (IgG1) monoclonal antibody conjugated to MMAE was shown to be well tolerated in a phase I trial (NCT01414283) of heavily pre-treated castrate resistant population [73]. Despite this study being primarily a dose-escalation trial, 14 (27%) patients had prostate-specific antigen (PSA) decreases of at least 25% and 6 (12%) had radiological improvements. Toxicities at the recommended phase II dose were mild and often transient, and included peripheral neuropathy, neutropenia and raised liver transaminases. The phase II single arm study of this PSMA ADC (NCT02020135) reported 14% of the 114 patients having PSA declines of > 50% and an ORR of 6%, suggesting some activity in a treatment refractory population [74]. Grade 3 or higher AEs did occur in 58% of patients, mainly haematological and there were 8 (7%) treatment related deaths. The greater toxicity seen in this study has made the future of this agent uncertain at this time. A similar patient population was involved in a phase I trial of an another PSMA targeting ADC carrying a pyrrolobenzodiazepine (PBD) payload namely MEDI3726 [75], and while clinical responses were seen at higher doses, TRAEs limited the duration of treatment, preventing further dose escalation and ultimately resulting in the study’s discontinuation. Ever since the very recent success of targeted radioligand therapy in this setting [76], numerous non-radioactive PSMA-targeting approaches are being actively researched, including chimeric antigen receptor T cells (CAR-T), vaccines and the promising bispecific T cell re-directed therapy [77]. Overall, although biologically appealing, the early clinical data pertaining to PSMA-targeting ADCs indicate modest efficacy at the cost of significant toxicity.

Other emerging antigen targets of interest in prostate cancer specific ADC development are STEAP1 and Trop-2. STEAP1 belongs to a family of transmembrane proteins and is upregulated selectively in prostate cancer, with its abnormal expression reportedly related to several oncogenic pathways [78]. DSTP3086S is a humanized IgG1 anti-STEAP1 monoclonal antibody conjugated to MMAE and was assessed in a phase I dose escalation study [79]. At the recommended phase II dose, 18% of the response-evaluable population experienced PSA decreases of > 50% and 6% had a radiological partial response. The transmembrane glycoprotein Trop-2 overexpressed in several cancers has also been found to be overexpressed in castrate resistant prostate cancer. In addition to promoting tumour progression and metastasis, it is associated with a poor prognosis and thought to be largely responsible for transdifferentiation into the occasionally seen aggressive neuroendocrine phenotype [80]. The Trop-2 targeting ADC SG is currently being investigated in a phase II study for patients with castration resistant prostate cancer progressing on anti-androgen therapy (NCT03725761). Another Trop-2 targeting ADC, DS-1062 (Daiichi Sankyo), which consists of a Trop-2 specific monoclonal antibody coupled with the topoisomerase inhibitor DXd, is yet to be investigated in prostate cancer, but is in early phase trials in NSCLC (NCT04484142, NCT04526691) and triple negative breast cancer (TNBC; NCT03401385) [81].

### RCC

cc-mRCC is a chemotherapy resistant tumour which is currently treated with ICIs, TKIs or both in combination [82, 83]. However, with several ICI-TKI combinations moving into earlier lines of treatment, there remains a need for novel agents in the refractory setting [84]. AGS-16C3F is a novel ADC targeting the ENPP3 antigen, whose expression is restricted in normal tissue but is high in RCC [85]. Patients with cc-mRCC progressing on two or more lines of treatment were eligible to receive either AGS-16C3F or the TKI axitinib in a phase II randomised clinical trial (NCT02639182) [86]. However, the primary endpoint of investigator assessed PFS was inferior (2.9 months *vs.* 5.7 months) with the ADC resulting in a negative trial.

TIM-1, a surface antigen which is also highly expressed in RCC, was the tumour target in a first-in-human phase I trial of CDX-014, a TIM-1 targeting IgG1 antibody linked to MMAE [87]. Patients with cc-mRCC following at least 2 lines of prior therapy were involved in the study, with 1 out of 16 patients (6%) exhibiting a partial response and another 5 (31%) experiencing disease stability on drug. There was one case of multiorgan failure resulting in treatment related death despite an overall manageable safety profile. Despite some early signal of activity, the developmental future of the drug is at this time unclear.

## ADCs in glioma

GBM, the most aggressive primary brain cancer in adults remains incurable and in desperate need of new treatment options [88, 89]. Clinical ADC development to date in GBM has been limited to EGFR targeting agents. A substantial body of evidence has demonstrated that overexpression of *EGFR*, as well as its tumour-restricted mutant form (EGFR variant III/vIII) plays key driver roles in GBM [90, 91]. Following several failed attempts at targeting EGFR in GBM using monoclonal antibodies and small molecule inhibitors, a number of novel ADCs that target the EGF family were tested. ABT-806 is a humanized antibody that selectively binds an epitope only rendered accessible in tumours with the EGFRvIII mutation or wild type EGFR amplification and thought to represent 45–50% of all adult GBM [92–94]. Depatuxizumab mafodotin (Depatux-M) composed of an antibody, ABT-806, targeting tumour specific activated EGFR linked to MMAF. Depatux-M was well tolerated and demonstrated signals of efficacy in early phase clinical trials [93, 95, 96]. In the phase II study of Depatux-M alone and with temozolomide *vs.* temozolomide or lomustine in recurrent EGFR amplified GBM (Intellance 2/EORTC 1410), patients with EGFR amplified GBM at first recurrence following standard chemoradiation were randomised to receive either Depatux-M alone, in combination with temozolomide (TMZ) or standard of care chemotherapy (lomustine/TMZ) [94]. While the trial did not meet its primary OS endpoint, longer follow-up demonstrated superior survival (HR 0.66 for combination *vs.* standard of care arm) in patients treated with the ADC-chemotherapy combination with the benefit most notable in those who relapsed later following chemoradiotherapy. In keeping with safety results from the phase I trial, ocular side effects (corneal epitheliopathy) were the most concerning toxicity associated with Depatux-M with grade 3 and 4 AE rates of 25–30%.

The phase III Intellance 1 trial (NCT02573324) tested Depatux-M *vs.* placebo in newly diagnosed EGFR-amplified GBM when administered concurrently with standard of care chemoradiotherapy. The primary endpoint was OS. Unfortunately, the trial was stopped due to an interim analysis confirming a lack of survival benefit and ultimately development of this agent has been halted [97].

AMG 595 is another EGFR targeting ADC consisting of a humanised antibody that binds solely to the EGFRvIII form of the receptor and not wild type EGFR, bound to the anti-tubulin agent DM1 via a noncleavable linker [98]. AMG 595 was studied in a phase I safety study enrolling patients with first or second recurrence of GBM and whose tumour demonstrated EGFRvIII expression by IHC [99]. In this subgroup of patients, 31 patients were response evaluable—2 (6%) experienced partial responses and 15 (47%) had disease stability, and the drug had an acceptable safety profile with grade 4 thrombocytopenia contributing to most dose-limiting toxicities (DLTs).

**Figure 1.**
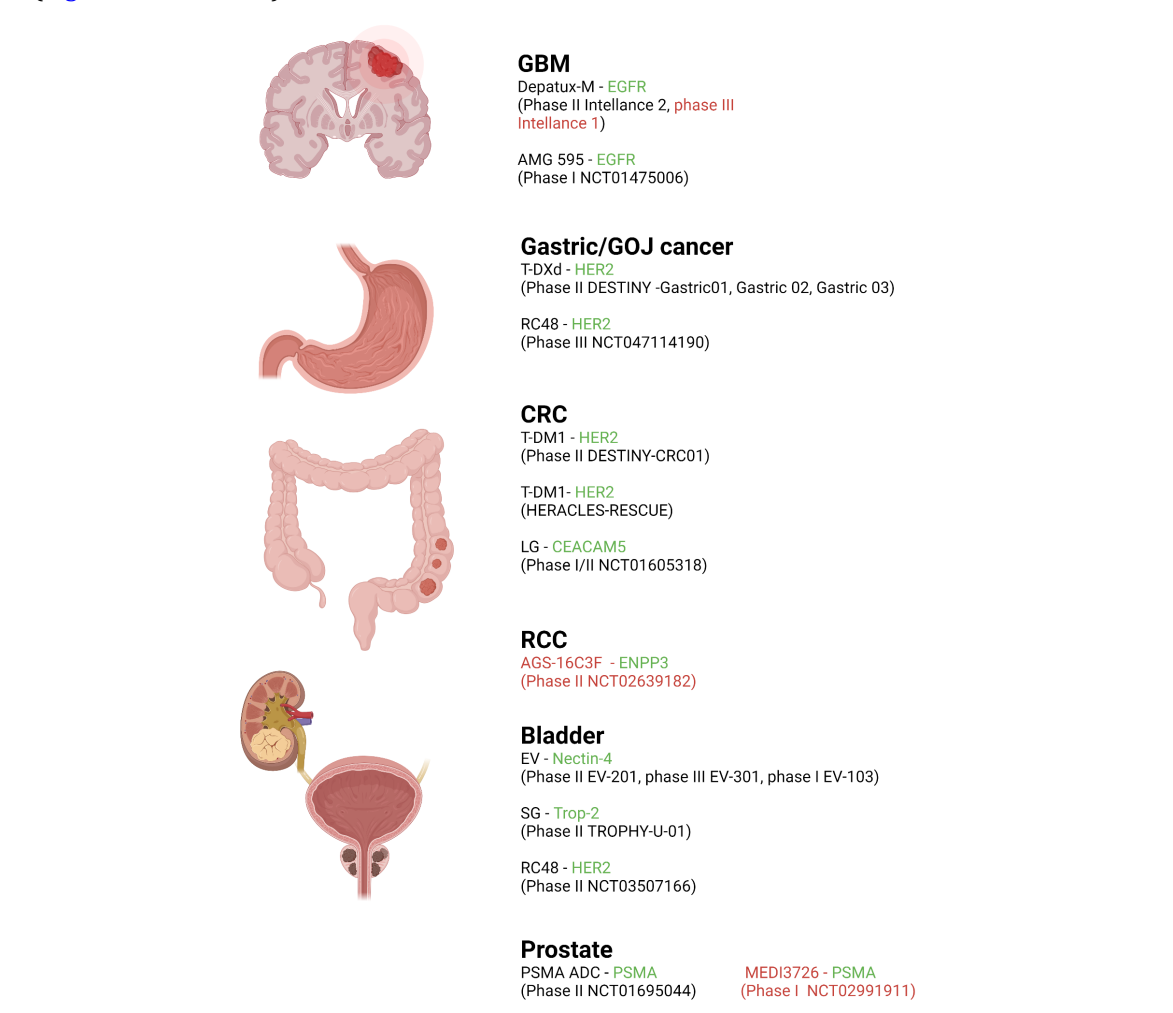
Recently completed and current active clinical ADC trials in select cancers (GBM, GOJ, CRC, RCC) including investigational agent and relevant trials. Antigen targets are shown in green and negative/discontinued trials are in red

A summary of the most recently developed, clinically relevant ADCs in GI and GU cancers and GBM, including their structure, target antigens, linkers, stage of development and FDA approval status is listed below (Figure 1 and Table 1).

**Table 1.** Summary of the most clinically relevant ADCs in selected tumour types

**Tumour type(s)**	**ADC**	**Target antigen**	**Antibody**	**Linker**	**Payload**	**Features and approvals**
GBM	Depatux-M/ABT-414	EGFR	Humanized IgG1	Non-cleavable MC linker	MMAF	Targets mutant EGFRvIII and tumours overexpressing wild-type EGFR [171]
GBM	AMG 595	EGFR	Human IgG1	Non-cleavable MCC linker	DM1	Targets only EGFRvIII and not wild-type EGFR [98]
Gastric, CRC	T-DXd/DS-8201	HER2	Humanized IgG1	Cleavable tetrapeptide-based linker	DXd (topoisomerase inhibitor/exatecan derivative)	High DAR of 8 (whilst maintaining stability), novel payload, effective across various histologies, bystander effect [172]. FDA breakthrough designation for GOJ cancer 2020
CRC, UC	RC48	HER2	Humanized IgG1 (hertuzumab)	Cleavable MC- valyl-citrullinyl-*p*-aminobenzyloxycarbonyl (MC-val-cit-PABC) linker	MMAE	Higher antibody affinity for HER2, more potent ADCC, bystander effect [39]
CRC	T-DM1	HER2	Humanized IgG1 (trastuzumab)	Cleavable tetrapeptide based (MCC) linker	DM1	First FDA approved ADC in solid organ tumours, vulnerable to drug efflux and resistance [173]
UC	SG	Trop-2	Humanized IgG (hRS7)	Cleavable maleimide based linker (CL2A)	SN-38 (irinotecan metabolite)	High DAR of 7.6, rapid clearance reducing off target toxicity [174]. FDA accelerated approval for UC 2021 [174]
UC	EV	Nectin-4	Humanized IgG1 (AGS-22M6)	Cleavable MC valine-citrulline linker	MMAE	FDA accelerated approval 2019 and breakthrough therapy designation with ICI in 2020 for la/mUC [58, 64, 175]
Prostate	PSMA ADC	PSMA	Human IgG1	Cleavable MC valine-citrulline linker	MMAE	Future development unknown [73, 74]
Prostate	MEDI3726	PSMA	Humanized IgG1	Cleavable valine-alanine linker	PBD	Development discontinued [75, 176]

ADCC: antibody-dependent cell-mediated cytotoxicity; MC: maleimidocaproyl; MCC: maleimidomethyl cyclohexane-1-carboxylate

## Emerging strategies and future directions

### Bispecific ADCs

Bispecific antibodies (bsAbs) are designed with the ability to bind two distinct antigens or two different epitopes belonging to the same antigen (also known as biparatopic antibodies) [100]. Many potential ADC targets either internalize poorly or undergo a high rate of endocytic recycling affecting payload delivery to the lysosomes [101]. The use of bispecific ADCs has the potential to enhance internalization by driving receptor clustering and cross-linking resulting in efficient trafficking to the lysosome and inhibiting recycling [102]. The prolactin receptor (*PRLR*) is expressed in a subset of breast cancers, and even at relatively low levels of expression, is constitutively internalised and rapidly trafficked to lysosomes for degradation, thus serving as a good therapeutic target for an ADC. A bsAb binding both HER2 and PRLR was conjugated to DM1 using a non-cleavable linker and showed superior *in vitro* activity compared to an “in house” generated HER2-DM1 ADC in breast cancer cell lines expressing both receptors [103]. Another bsAb-ADC, consisting of an antibody binding both HER2 and the trafficking protein CD63 conjugated to a microtubule-disrupting payload called duostatin-3 was found to be more potent in HER2-positive breast xenografts than the HER2 and CD63 ADCs as monotherapy [104]. A biparatopic ADC consisting of an antibody construct capable of binding two different epitopes (subdomains 2 and 4 of the ectodomain) on HER2 and conjugated to the tubulysin (Tub) AZ13599185 showed more anti-tumour activity than TDM-1 in trastuzumab resistant tumour models as well as demonstrating activity in TDM-1 resistant and HER2-low xenografts. The success of this agent was attributed to several factors including more efficient cytotoxin delivery by redirection of HER2 from recycling to lysosomal degradation, a payload that avoided the DM1 efflux pumps, inhibition of ligand dependent signalling and enhanced bystander effect [102]. Based on these data, a phase I dose escalation trial of the biparatopic ADC (MEDI4267) followed in treatment refractory HER2-positive breast and gastric cancers [105]. The trial reported limited clinical activity, high toxicity rates and an unfavourable pharmacokinetic profile, all of which makes further clinical development unlikely. Additional trials are awaited of other bsAb’s and related ADCs to determine whether their preclinical promise will actually deliver.

### The TME—a promising area for ADC development

The TME is a complex and dynamic system consisting of stromal and immune cells, as well as an extracellular cellular matrix (ECM), which serves as mechanical scaffolding as well as a source of key growth factors and signalling molecules implicated in cancer development [106–108]. The stromal compartment consists of a collection of non-tumour host derived cells such as fibroblasts, endothelial cells, myeloid cells such as tumour associated macrophages (TAMs) and myeloid-derived suppressor cells (MDSCs) together with cells representing the innate (natural killer cells, dendritic cells) and adaptive immune systems (cytotoxic T cells) [109]. The TME may comprise up to 50% of the tumour mass and due to its critical role in tumorigenesis as well as resistance to certain therapeutics has been garnering interest as a potential therapeutic target (Table 2) [106, 110].

**Table 2.** Successful recent preclinical studies of TME targeting ADCs

**Target**	**TME component**	**ADC**	**Payload**	**Tumour types**	**Translation to clinic**
LRRC15	CAF	ABBV-085	MMAF	NSCLC, osteosarcoma, breast cancer, GBM	Phase I trial in sarcoma and other advanced solid tumours (published 2021) [113]
FAPα	CAF	OMTX705 (+ ICI)	TAM470 (MTI)	NSCLC, TNBC, ovarian, pancreatic	Pending
TEM8	Stroma	m825-MMAE	MMAE	CRC, breast, lung, ovarian, pancreatic	Pending
TF	Stroma	TF antibody-MMAE	MMAE	pancreatic	Phase II trial (TV) in refactaory cervical cancer—positive (published 2021) [127]
EphA3	Stroma	IIIA4-USAN	USAN	GBM	Pending

CAF: cancer associated fibroblast; Eph: erythropoietin-producing hepatocellular carcinoma; FAPα: fibroblast activating protein α; LRRC15: leucine-rich repeat containing 15; MTI: microtubule inhibitor; TEM8: tumour endothelial marker 8; TF: tissue factor; TV: tisotumab vedotin; USAN: maytansine; +: plus

Targeting cells such as CAFs has been particularly appealing in stroma rich cancers such as pancreatic cancer, TNBC and sarcoma [111–113]. LRRC15 is a type 1 membrane protein with no obvious intracellular signalling domain that has been found to be overexpressed on CAFs and mesenchymal stem cells (MSCs) in several solid tumours [114]. A novel ADC consisting of a humanized IgG1 antibody targeting LRCC15 conjugated to MMAE via a protease cleavable linker, ABBV-085, when tested in preclinical models with variable LRCC15 expression demonstrated superior tumour growth inhibition (TGI) including in tumours negative for LRCC15 expression. However, when the MMAE payload of ABBV-085 was substituted by the structurally similar, but non-cell-permeable payload MMAF, there seemed to be no anti-tumour activity in the cancer-negative/stromal-positive models in *vivo*, despite having similar *in vitro* efficacy to ABBV-085. Thus, the cell kill of adjacent cancer cells from the “bystander effect” by the permeable MMAE toxin, following uptake and processing by the CAFs, could be an important complementary mechanism of action for this ADC in addition to direct CAF killing. Finally, studies exploring the combination of ABBV-085 with various anticancer therapies including targeted therapy, chemotherapy and radiation also showed potent anti-tumour activity, yielding 100% CRs in 2 out of 4 models—ABBV-085 with erlotinib in lung adenocarcinoma and with gemcitabine in squamous cell carcinoma. The encouraging ABBV-085 preclinical data have resulted in the first-in-human phase I trial (NCT02565758) assessing safety and activity of the ADC in sarcomas and other solid tumours [115].

FAPα, a cell surface protein expressed on activated CAFs in many tumour types, is considered to be a crucial contributor to the immunosuppressive TME and is associated with a poor prognosis [116, 117]. Despite promising preclinical data, anti-FAP antibodies and small-molecule inhibitors targeting FAP failed to show clinical benefit in clinical trials [118, 119]. OMTX705 is a novel ADC generated by combining a humanized IgG1 anti-FAPα monoclonal antibody (OMTX005) with a novel MTI TAM470. Treatment with OMTX705 resulted in TGI of close to 100% in patient-derived xenograft (PDX) models of lung, triple negative breast, ovarian and pancreatic cancers [120] as monotherapy, as well as producing synergistic and durable responses when combined with chemotherapy. In pembrolizumab resistant NSCLC PDX model, OMTX705 demonstrated substantial tumour regression and when combined with pembrolizumab, resulted significant anti-tumour activity with durable responses as well as CRs seen. *Ex vivo* analysis of the tumours treated with both ADC and programmed cell death protein 1 (PD-1) inhibitor confirmed a more robust influx of CD8^+^ T cells, possibly explaining the success of the combination over single agent activity, and the deeper responses seen in immunocompetent models as opposed to immunodeficient ones. Additionally, these results are consistent with our understanding of CAFs and their role in suppressing anti-tumour immunity through modulation of primarily tumour necrosis factor α (TNF-α) and interferon γ (IFN-γ) [120].

Another novel target for ADC development has been TEM8, a transmembrane glycoprotein initially found to be a TEM but more recently also shown to be expressed widely in the tumour stroma across several human tumours [121–123]. The TEM8 targeting m825 [124] was developed into an ADC by conjugating it with MMAE via a cleavable linker. *In vivo* efficacy was tested in immunodeficient nude mice in a range of tumour models including colon, breast, lung, ovarian and pancreatic tumours. m825-MMAE showed superior TGI compared to chemotherapy, with the combination of ADC and chemotherapy outperforming both drugs as monotherapy. The role of stroma targeting was elucidated further by using TEM8 negative cell lines in transgenic mouse models; the tumour inhibition was significantly higher in TEM8 wild type than in TEM8 knockout mice, indicating targeting TEM8 was required for a therapeutic effect. Importantly, extensive *ex vivo* and supporting in *vitro* work on the treated tumours revealed that while there was binding of the ADC to TEM8^+^ stromal cells, it was tumour cell apoptosis that seemed to elicit the tumoricidal responses. Hence, the bystander effect in this case was driven by drug activation and release through the stroma.

TF, a transmembrane glycoprotein well known for its role in the extrinsic coagulation pathway has also been found to be overexpressed in several cancers [125], and therefore an appealing target for anti-cancer drug development. In addition to its presence on cancer cells, TF expression has also seen to be upregulated in stromal components of the TME including endothelial cells and fibroblasts [126, 127]. Based on this, an evaluation of a mouse TF-targeting ADC in the clinically relevant *LSL-Kras^G12D/+^*; *LSL-Trp53^R172H/+^*; *PDX-1-Cre* (KPC) model of pancreatic adenocarcinoma was undertaken [128]. A pancreatic cancer cell line was established from KPC mice and transfected the cells with the mouse *TF* gene. Established tumours thus had high cell and stromal expression of TF. The ADC generated linking the anti-mouse TF antibody to MMAE using a bis-alkylating conjugation method demonstrated significant TGI in orthotopic and subcutaneous pancreatic cancer allografts. It is of note that this tumour model recapitulates the stroma rich TME of pancreatic adenocarcinoma. Although, tumour regressions were not seen, the studies did demonstrate an OS benefit in the mice treated with the ADC *vs.* vehicle control and control ADC. Further analysis of treated tumours revealed a dual inhibition mechanism of action, as evidenced by apoptosis of both cancer and stromal cells particularly vascular endothelial cells. Of note, the TF-targeting ADC TV was evaluated in the phase II efficacy and safety of TV in previously treated recurrent or metastatic cervical cancer (innovaTV 204) trial [129], which yielded an ORR of 24%, disease control rate (DCR) of 72% and an mDOR of 8.3 months (4.2 months–not reached). Based on these findings a biologics license application seeking accelerated approval for the management of recurrent or metastatic cervical cancer has been submitted to the FDA.

The Eph receptors and their cell-associated ephrin ligands have been implicated in the growth and progression of a large range of cancers and are increasingly recognized as important therapeutic anti-cancer targets [130]. EphA3, a member of the Eph family of receptors, has been found to be overexpressed in several cancers with roles in oncogenesis, poor survival, and response to therapies [131–134]. Whilst their expression as target antigens on tumour cells has inspired active drug discovery research [133, 135, 136], it is their presence in the stromal TME that offers an appealing and novel anti-cancer approach. Having determined the preferential expression of EphA3 on tumour adjacent bone marrow-derived mesenchymal (BMSCs), myeloid and vascular cells, the effect of a chimeric EphA3 agonistic antibody (chIIIA4) was investigated in mouse xenograft models of colon and prostate carcinoma [137]. ChIIIA4 treatment resulted in significant TGI through reduction of MSCs and successful disruption of the tumour vasculature. Direct EphA3 targeting in the form of small molecule kinase inhibitors and monoclonal antibodies have progressed to early phase clinical trials both in hematological and solid (GBM) cancers [136, 138]. Based on this, Offenhäuser et al. [135] successfully demonstrated that the EphA3 antibody IIIA4 conjugated with the cytotoxic microtubule-targeting agent USAN was effective in inhibiting tumour growth in orthotopic xenograft models of glioma. Given the integral role played by the TME in glioma development and that the EphA3 receptor has been found to be expressed on both MSCs as well as tumour infiltrating cells of bone marrow origin (macrophages/microglia) in this disease [139], targeting this niche using appropriate drug conjugates offers an attractive therapeutic option. However, further preclinical research into EphA3 binding ADCs for gliomas is indeed required in a cancer of dire need.

### ADCs in combination with immune and other therapies

The landscape of early drug development and cancer therapeutics is evolving, as it is being increasingly recognised that in fact combinatorial strategies involving more than one agent with varying mechanisms of action are perhaps more likely to induce greater and more durable anti-tumour responses, as well as reduce the chances of acquired treatment resistance [140, 141]. Combining ADCs with chemotherapy (ideally with a mechanism of action different to the payload) has the theoretical advantage of avoiding clonal resistance, as evidenced by the TEM8 specific ADC discussed previously [142]. Similarly, combinations of ADCs with small molecule kinase inhibitors, and other antibody-based treatments such as anti-VEGF agents and ICB are all appealing opportunities to augment ADC efficacy and have demonstrated successful synergy in several preclinical studies [143]. Of these strategies, the addition of IO agents, particularly ICIs to molecularly targeted agents such as ADCs has garnered the most attention and promises the most potential [141, 144, 145].

The surge in interest in combining ADCs with immunotherapeutics requires understanding the effect of the host immune response on the drug-tumour interaction paramount. There is evolving pre-clinical and some preliminary clinical evidence that points to a favourable synergy between the two drug classes based on the critical role of the adaptive immune system [146]. As discussed previously, the FAPα binding ADC OMTX705 when combined with an ICI proved to be a potent combination owing to the resulting influx of CD8 effector cells. Biopsies retrieved 3 weeks after exposure to the HER2 targeting ADC TDM-1 in the adjuvant dynamic marker-adjusted personalized therapy optimising risk assessment and therapy response prediction in early breast cancer (WSG-ADAPT) trial demonstrated an increase in the number and density of tumour infiltrating T cells [147, 148]. This was further explored in a trastuzumab-resistant HER2-expressing syngeneic mouse model, which enabled a focused evaluation of the immunological and therapeutic impact of the cytotoxic payload [147]. The orthotopically grown tumours in transgenic mice overexpressing human *HER2* treated with TDM-1 were found to have an increase in tumour-infiltrating lymphocytes (TILs), specifically T-lymphocytes. Further analysis of the lymphocytes and TAMs in the treated tumour specimens revealed increased levels of the inhibitory molecule cytotoxic T-lymphocyte-associated antigen 4 (CTLA-4) on CD4^+^ and CD8^+^ lymphocytes as well as programmed death ligand-1 (PD-L1) on TAMs when compared to the pre-treatment samples. Furthermore, while the combination of CTLA-4 and PD-1 blocking antibodies did not have antitumour effects by themselves, combining them with TDM-1 resulted in potent anti-tumour activity yielding a near 100% cure rate [147]. Hence these data suggest that T-DM1 treatment promoted a T cell-inflamed tumour phenotype with a higher density of tumour infiltrating T cells and an increased chemokine profile as well as an upregulation of the molecular targets for checkpoint inhibition, which in turn translated into the successful anti-tumour activity. The combination of atezolizumab with TDM-1 was evaluated as part of the phase Ib GO29381 trial (NCT02605915) and did confirm safety of the combination as well as evidence of activation of the adaptive immune system upon subsequent biomarker analysis [149]. However, in the phase II study that followed, the addition of atezolizumab to T-DM1 in patients with metastatic HER2 positive breast cancer previously treated with trastuzumab and taxane did not demonstrate a PFS benefit. Additionally, the combination was associated with more AEs although there appeared to be some treatment benefits in the PD-L1 positive patients [150]. With respect to next generation ADCs, trials are currently ongoing involving T-DXd and ICIs (Table 3).

**Table 3.** Currently recruiting and recently completed trials exploring the combination of ADC and IO agents

**Phase**	**Trial**	**Organ system**	**ADC**	**Antibody target**	**Payload (class)**	**IO agent (mechanism of action)**	**Status**
I	NCT03523572	Breast and urothelial	T-DXd	HER2	DXd (DDA)	Nivo (ICI)	Recruiting (2018)
I	NCT04042701	NSCLC, breast	T-DXd	HER2	DXd (DDA)	Pembrolizumab (ICI)	Recruiting (2020)
I	NCT03364348	Breast	T-DM1	HER2	DM1 (TI)	Utomilumab (CSA)	Recruiting (2017)
I	NCT03032107	Breast	T-DM1	HER2	DM1 (TI)	Pembrolizumab (ICI)	Recruiting (2017)
II	NCT02924883	Breast	T-DM1	HER2	DM1 (TI)	Atezolizumab (ICI)	Published 2021-negative trial
I	NCT02341625	Solid organ, mesothelioma	BMS-986148	Mesothelin	Unknown	Nivo (ICI)	Active (2015). Not recruiting
I	NCT03816358	Pancreatic	Anetumab ravtansine	Mesothelin	DM4 (TI)	Nivo + ipilimumab/Nivo + chemo (ICI)	Recruiting (2019)
I	NCT03455556	NSCLC	Anetumab ravtansine	Mesothelin	DM4 (TI)	Atezolizumab (ICI)	Completed (2018–2020)
I	NCT03126630	Mesothelioma	Anetumab ravtansine	Mesothelin	DM4 (TI)	Pembrolizumab	Recruiting (2017)
I	NCT04448886	HR^+^/HER2-breast	SG	Trop-2	SN38 (TI)	Pembrolizumab (ICI)	Recruiting (2020)
II	NCT04468061	TNBC	SG	Trop-2	SN38 (TI)	Pembrolizumab	Recruiting (2020)
Ib/II	NCT03288545	Urothelial	EV	Nectin-4	MMAE (TI)	Pembrolizumab (ICI)	Recruiting (2017)—FDA breakthrough designation 2019 [177]
I	NCT03310957	Breast	Ladiratuzumab vedotin	LIV-1 (zinc transporter subfamily)	MMAE (TI)	Pembrolizumab (ICI)	Recruiting (2018)
I	NCT03000257	SCLC	Rovalpituzumab tesrine	DLL3	SC-DR002 (DDA)	Budigalimab (PD-LI)	Completed (2019)
I/II	NCT03026166	SCLC	Rovalpituzumab tesirine	DLL3	SC-DR002 (DDA)	Nivo + ipilimumab (ICI)	P1 completed (2018), P2 halted (2019) due to high number of DLTs [178]
I	NCT03639194	SCLC	SC-011	Unknown	DDA	ABBV-181 (ICI)	Recruiting (2018)
I	NCT02099058	NSCLC, solid tumours	Telisotuzumab vedotin	*cMET*	MMAE (TI)	Nivo	Recruiting (2014)
I	NCT03786081	Cervical	TV	TF	MMAE (TI)	Pembrolizumab	Recruiting (2019)
I	NCT03729596	Advanced solid tumours	MGC018	Anti-B7-H3	Unknown	MGA012 (ICI)	Recruiting (2018)
II	NCT03835819	Endometrial	Mirvetuximab soravtansine	FRα	DM4 (TI)	Pembrolizumab	Recruiting (2019)

*cMET*: mesenchymal epithelial transition factor; CSA: co-stimulatory agent; DDA: DNA damage agent; DLL3: delta-like ligand 3; FRα: folate receptor alpha; HR^+^: hormone receptor positive; LIV-1: zinc transporter ZIP6/SLC39A6; Nivo: nivolumab; TI: tubulin inhibitor; +: plus

The novel anti-HER3 ADC (patritumab DXd; U3-1402) consisting of the anti-HER3 antibody patritumab conjugated with the topoisomerase isomerase inhibitor DXd as the toxin payload has been investigated as monotherapy in a safety study of patients with EGFR inhibitor resistant, EGFR-mutated lung cancer [151] and is currently being evaluated in patients with HR^+^/HER negative early breast cancer (NCT04610528) [152]. Preclinically, U3-1402 was evaluated in combination with PD-1 inhibition in syngeneic HER3 overexpressing tumour models [153]. Following establishment of a syngeneic mouse model by subcutaneous inoculation of an HER3 overexpressing mouse melanoma cell line and successful tumour inhibition by the ADC, extensive evaluation of mechanisms of anti-tumour immunity were undertaken. Despite a similar number of CD8^+^ TILs in both treatment and control arms, there was a decrease in the expression of inhibitory molecules such as PD-1, lymphocyte activation gene-3 (*LAG3*) and T cell immunoglobulin and mucin-domain containing molecule-3 (TIM-3) on the CD8^+^ lymphocytes. This was in contrast to the T-DM1 study which in fact found increase in the inhibitory molecules following treatment with the HER2 ADC. However, consistent with the TDM-1 experiments, tumours treated with U3-1402 exhibited higher levels of pro-inflammatory cytokines such as IFN-γ, TNF-α, and IL-2, and *in vivo* depletion of CD8^+^ cells reduced its anti-tumour efficacy. The combination of U3-1402 with anti-PD-1 therapy also resulted in augmented responses, even in tumours resistant to PD-1 treatment alone.

Antibodies targeting one of the Eph family receptors, ephrin type-A receptor 2 (EphA2) loaded with PBD and Tub payloads were used to perform a thorough evaluation of their immunomodulatory effects [154]. Initially, a vaccination/challenge assay using the colon cancer CT26 cell line treated with either Tub, methylmep-*N*-ethyl-tubulysin aniline (MMETA) or PBD, demonstrated successful protection against subsequently injected CT26 cells in 40% and 70% of mice respectively suggesting these payloads could elicit immunological cell death (ICD) on their own. *In vivo* efficacy studies involved treatment of CT26 and MCA205 (a weakly immunogenic fibrosarcoma derived cell line) tumour bearing mice with the EphA2-PBD or EphA2-Tub conjugates, resulting in a large percentage of CRs. Subsequently, tumour rejection rates were high in the mice that had experienced a CR when the cell lines were re-introduced, suggesting an element of tumour-specific immunological memory. Both ADCs were more potent when tested in immunocompetent mouse models compared to immunodeficient hosts and CD8^+^ depletion using an anti-CD8 antibody significantly abrogated the efficacy of the ADCs in the CT26 model, suggesting a crucial role once again for effector T-lymphocytes in mediating anti-tumour activity. These observations confirmed the phenomena of ICD, immunological memory, and optimal T cell regulation when using ADCs. Finally, the combination of EphA2 ADCs with immunotherapies such as PD-L1 inhibitors and agonists of the glucocorticoid induced TNF receptor-related protein (GITR) in the CT26 model resulted in dramatic anti-tumour responses once again lending credence to potential for effective synergy.

While ICB and targeted therapy against oncogenic mutations have yielded great therapeutic successes in cancers such as lung and melanoma, resistance to treatment remains a challenge [155]. One mechanism of resistance that has been identified is the activation of the tyrosine-protein kinase receptor UFO (AXL) possibly via its role in epithelial-to-mesenchymal transition [156, 157]. Building on previous research demonstrating successful tumour inhibition using an AXL-targeting ADC across several AXL expressing tumour types [158], further research to better characterise the anti-tumour immune response associated with the ADC was undertaken using enapotamab vedotin (EnaV). This AXL targeting antibody conjugated to MMAE was evaluated in AXL-high mouse models of melanoma and lung cancer and found to produce an inflammatory microenvironment characterised by an influx of T cells with a skew to lymphocytes with a memory-like phenotype (CD137-high) [158]. Perhaps most notably, EnaV demonstrated significant anti-tumour activity in cancer models that were resistant to ICB as well as offering *de novo* sensitivity to ICB in tumour models otherwise insensitive to PD-1 inhibition.

Despite HER2, HER3, EphA2 and AXL involving distinct receptor tyrosine kinase (RTK) targets, varying payloads and demonstrating subtle differences in the modulation of the tumour immune microenvironment (TIME), they all clearly show induction of an inflamed, T cell rich TME which facilitated synergy between ADCs and immunotherapy in animal models. The favourable data from these pre-clinical studies and others have inspired a large number of clinical trials involving combinations of various ADC’s and IO agents which are ongoing (Table 3). A recent success based on this synergy has been the combination of the Nectin-targeting ADC, EV and the ICI pembrolizumab which have been examined in the first line setting, with preliminary results of the phase I EV-103 trial of the urothelial cohort updated this year [159, 160]. The combination of EV and pembrolizumab demonstrated encouraging results with an ORR of 73%, a DCR of 93% and an mDOR of 25 months. The combination was found to be safe and manageable, with relatively low rates of clinically relevant TRAEs such as peripheral neuropathy (≥ grade 3, 4%) and fatigue (≥ grade 3, 11%).

## Conclusions

ADCs clearly have had a major impact on oncology in the last decade thus spurring on development in multiple cancer types. However, it will be important to ensure that this is done, in both an efficient and rigorous manner, given the multitude of ADCs that will require testing with a range of different targets, payloads and linkers. In 2021, cancers of the upper GI tract have had a landmark year in terms of new FDA approvals for ICIs. Checkpoint inhibitors have received approvals both as single agent therapy in the adjuvant setting [161] as well as in combination with chemotherapy [162] and trastuzumab in advanced tumours [163]. In contrast, CRC remains relatively unresponsive to ICIs, with the exception of microsatellite-instability-high tumours [164]. The promise shown by T-DXd has reinvigorated interest in HER2 targeting using ADCs in cancers of the upper and lower GI tract, although the most appropriate line of treatment and its potential role in combination with other agents remains to be clarified. Other surface antigens such as CEACAM5 do possess appeal as therapeutic targets for ADCs, although further clinical research is warranted. With respect to GU cancers, UC and ccRCC have witnessed several approvals for ICIs, and in the case of ccRCC newer TKIs as well [165–169]. The approval of the ADCs EV and SG in advanced UC represents yet another distinct and valuable drug class which has proven effective in chemoimmunotherapy refractory settings. Once again, their roles as combination partners to other standard of care treatments are yet to be confirmed despite favourable early signals from the combination of EV and pembrolizumab. Progress in ADC development has been slower in ccRCC and prostate cancer and its position in these cancers is currently unclear. The EGFR targeting Depatux-M demonstrated signals of efficacy in phase I and II trials, perhaps paving the way forward for further development of ADCs in GBM, a disease in dire need of treatment options. Outside of direct tumour cell targeting in GBM, the exploitation of its critical TME using ADCs designed against stromal components could be an avenue worth exploring, such as with EphA3 binding agents.

Preclinically appealing targets may require significant clinical testing and development to confirm therapeutic efficacy in the clinic. ADCs such as T-DXd and SG have shown activity and even achieved regulatory approvals across different tumour histologies. It will be necessary to test new ADCs in in each cancer type as there are also data that the tumour context can result in vastly different efficacy or toxicity with the same drug. This likely contributes to the trend for a transition from “all comer” phase I trials to basket trials in the ADC space [170]. Lastly, it is very likely that maximal benefit for patients will require combinatorial methods. Whilst combinatorial ADC approaches to enhance tumour directed killing with minimal toxicity are one attractive strategy, there is also a strong rationale to combine ADCs with other treatment modalities, including relatively tumour-agnostic approaches such as combination with chemotherapy, radiotherapy or drugs targeting the TME or immune system. We anticipate and look forward to future successes with ADCs for cancer treatment in multiple tumour types and their increasing integration into standard of care treatment regimens.

## Abbreviations

ADCs: antibody-drug conjugates
AEs: adverse events
bsAbs: bispecific antibodies
CAF: cancer associated fibroblast
ccRCC: clear cell renal cell carcinoma
CEA: carcinoembryonic antigen
CEACAM5: carcinoembryonic antigen-related cell adhesion molecule 5
CI: confidence interval
CR: complete response
CRCs: colorectal cancers
DAR: drug to antibody ratio
DDA: DNA damage agent
Depatux-M: depatuxizumab mafodotin
DM1: drug maytansinoid 1
DXd: deruxtecan
EGFR: epidermal growth factor receptor
ENPP3: ectonucleotide pyrophosphatase/phosphodiesterase 3
Eph: erythropoietin-producing hepatocellular carcinoma
EphA2: ephrin type-A receptor 2
EV: enfortumab vedotin
FAPα: fibroblast activating protein α
FDA: Food and Drug Administration
GBM: glioblastoma
GI: gastrointestinal
GOJ: gastro-oesophageal junction
GU: genitourinary
HER2: human epidermal growth factor 2 receptor
HERACLES: human epidermal growth factor 2 receptor amplification for colo-rectal cancer enhanced stratification trial
HR: hazard ratio
ICB: immune checkpoint blockade
ICIs: immune checkpoint inhibitors
IgG1: immunoglobulin G1
IHC: immunohistochemistry
ILD: interstitial lung disease
IO: immuno-oncology
la/mUC: locally advanced or metastatic urothelial cancer
LG: labetuzumab govitecan
MC: maleimidocaproyl
mCRC: metastatic colorectal cancer
mDOR: median duration of response
MMAE: monomethyl auristatin E
MSCs: mesenchymal stem cells
Nivo: nivolumab
NSCLC: non-small cell lung cancer
ORRs: objective response rates
OS: overall survival
PBD: pyrrolobenzodiazepine
PD-1: programmed cell death protein 1
PD-L1: programmed death ligand-1
PDX: patient-derived xenograft
PFS: progression free survival
PSA: prostate-specific antigen
PSMA: prostate-specific membrane antigen
RC48: disitamab vedotin
RCC: renal cell carcinoma
SCLC: small cell lung cancer
SG: sacituzumab govitecan
STEAP1: six-transmembrane epithelial antigen of the prostate 1
TAMs: tumour associated macrophages
T-DM1: ado-trastuzumab emtansine
T-DXd: trastuzumab deruxtecan
TEM8: tumour endothelial marker 8
TF: tissue factor
TGI: tumour growth inhibition
TI: tubulin inhibitor
TIM-1: T cell immunoglobulin mucin-1
TKIs: tyrosine kinase inhibitors
TME: tumour microenvironment
TNBC: triple negative breast cancer
TNF-α: tumour necrosis factor α
TRAEs: treatment related adverse events
Trop-2: trophoblast cell surface antigen 2
Tub: tubulysin
TV: tisotumab vedotin
UC: urothelial cancer
USAN: maytansine
VEGF: vascular endothelial growth factor

## Declarations

### Copyright

© The Author(s) 2022.
